# Maternity care providers’ perceptions of women’s autonomy and the law

**DOI:** 10.1186/1471-2393-13-84

**Published:** 2013-04-04

**Authors:** Sue Kruske, Kate Young, Bec Jenkinson, Ann Catchlove

**Affiliations:** 1 Queensland Centre for Mothers & Babies, The University of Queensland, Brisbane, QLD, 4067, Australia

**Keywords:** Autonomy, Decision making, Legal accountability, Maternity care, Childbirth, Maternal rights, Fetal rights, Health professionals

## Abstract

**Background:**

Like all health care consumers, pregnant women have the right to make autonomous decisions about their medical care. However, this right has created confusion for a number of maternity care stakeholders, particularly in situations when a woman’s decision may lead to increased risk of harm to the fetus. Little is known about care providers’ perceptions of this situation, or of their legal accountability for outcomes experienced in pregnancy and birth. This paper examined maternity care providers’ attitudes and beliefs towards women’s right to make autonomous decisions during pregnancy and birth, and the legal responsibility of professionals for maternal and fetal outcomes.

**Methods:**

Attitudes and beliefs around women’s autonomy and health professionals’ legal accountability were measured in a sample of 336 midwives and doctors from both public and private health sectors in Queensland, Australia, using a questionnaire available online and in paper format. Student’s *t*-test was used to compare midwives’ and doctors’ responses.

**Results:**

Both maternity care professionals demonstrated a poor understanding of their own legal accountability, and the rights of the woman and her fetus. Midwives and doctors believed the final decision should rest with the woman; however, each also believed that the needs of the woman may be overridden for the safety of the fetus. Doctors believed themselves to be ultimately legally accountable for outcomes experienced in pregnancy and birth, despite the legal position that all health care professionals are responsible only for adverse outcomes caused by their own negligent actions. Interprofessional differences were evident, with midwives and doctors significantly differing in their responses on five of the six items.

**Conclusions:**

Maternity care professionals inconsistently supported women’s right to autonomous decision making during pregnancy and birth. This finding is further complicated by care providers’ poor understanding of legal accountability for outcomes experienced in pregnancy and birth. The findings of this study support the need for guidelines on decision making in pregnancy and birth for maternity care professionals, and for recognition of interprofessional differences in beliefs around the rights of the woman, her fetus and health professionals in order to facilitate collaborative practice.

## Background

All health consumers, including pregnant women, have the right to make autonomous decisions about their medical care. This includes the ability to decline to follow medical advice, guidelines or policy [1]. Maternity care is distinct from other areas of healthcare in that pregnancy is not an illness but rather a normal physiological process. Furthermore, the interdependent relationship between the mother and the fetus raises unique legal and ethical concerns [2]. As such, the seemingly clear principle of autonomy has created confusion and tension for a number of maternity care stakeholders including women, care providers, policy makers and insurers [2-4].

Maternity care stakeholders, (e.g., clinicians, policy makers, insurers) can influence how pregnant women’s rights during pregnancy and birth are upheld. However, care providers (e.g., midwives, obstetricians) generally have the most contact with pregnant women and as such can significantly influence women’s ability to exercise their legal rights [2]. It is therefore important to understand care providers’ perceptions of women’s autonomy and the rights of the fetus in order to inform clinical practice as well as to support women to understand and exercise their right to autonomy during pregnancy and birth.

While there is a body of work focussed on the related concept of informed consent [5,6] and on the extent to which women’s rights to autonomy are upheld in the provision of maternity services [7-9], there is no published work where the focus is on care providers perceptions of the ethico-legal principle of autonomy. The barriers and enablers to women’s involvement in decision making as part of a suite of outcomes have also been examined [10-12], but these studies do not appear to extend to specific consideration of autonomy or the experiences or beliefs of women or care providers when requested care is at variance to professional advice.

This paper reports on maternity care providers’ attitudes and beliefs towards women’s right to make autonomous decisions during pregnancy and birth, and the legal responsibility of professionals for both maternal and fetal outcomes.

## Method

This study formed part of a larger study on interprofessional collaboration reported elsewhere [13].

### Materials and procedure

A web-based survey was the predominant method of data collection. Paper-based surveys were also distributed to professionals with publicly available postal addresses. Data collection occurred between February and May 2010. A pilot study, interviews, and expert feedback were conducted in order to enhance content validity. Participants were asked to forward the web-based survey link or photocopy the paper-based survey for other colleagues to complete.

The survey measured professionals’ attitudes and beliefs about collaboration in maternity care. Items were developed around the following domains: definition of collaborative practice; current workplace practice; models of care; factors affecting collaborative practice; professional values and beliefs; and collaborative practice in Queensland (see Additional file 1 for complete questionnaire). Six items were of interest to this paper. These measured care providers’ attitudes towards various components of medical models of care, woman-centred care, and the current maternity care system. These included decision making, women’s autonomy, and legal responsibility for outcomes of birth (see Table 1 for items asked). Participants indicated their agreement to each item on a 7-point Likert scale (1 = *strongly disagree* to 7 = *strongly agree,* with a midpoint of 4 = *neither agree nor disagree*).

**Table 1:** ***T*-test results comparing midwives and doctors’ responses for each survey item**

**Item**	**Midwives**	**Doctors**	***t***	***p***	**95% CI**		***η***^**2**^
	***M (SD)***	***M (SD)***			***LL***	***UL***	
In collaborative practice, working with primary carers, the final decision should always rest with the woman	5.72 (1.19)	4.82 (1.65)	−3.87	.001***	−1.37	-.44	.04
Collaboration involves midwives and doctors working together but the doctor is the most competent in making the final decision	2.54 (1.55)	4.95 (1.42)	11.29	.001***	1.98	2.82	.28
For the safety of the baby, the maternity care team sometimes need to override the needs of the woman	4.27 (1.77)	4.89 (1.48)	2.71	.008	0.17	1.08	.02
Encouraging women to have more control over their childbearing compromises safety	2.14 (1.30)	3.22 (1.33)	5.61	.001***	0.70	1.46	.08
Legally, doctors are ultimately responsible, even in collaborative models	2.69 (1.57)	5.75 (1.34)	15.04	.001***	2.65	3.46	.04
The current maternity care system allows all to be legally accountable for their own actions in a collaborative team	4.21 (1.61)	3.13 (1.40)	−4.61	.001***	−1.54	−0.62	.06

*Note.* Bonferroni adjustment of .008. *CI* = confidence interval; *LL* = lower limit, *UL* = upper limit.

***p < .001.

This study followed normal ethics protocol. There was no incentive given to complete the survey and no confidential data were received. Completion of the survey was taken as an indicator of consent to participate. Ethics approval was obtained from the University of Queensland Medical Research Ethics Committee (Consent No. 2009001651).

### Data analysis

All analyses were conducted using SPSS Statistics version 20.0. Obstetricians and General Practitioner (GP) obstetricians did not differ in their responses on any of the items examined. Therefore, in order to improve power and to compare the responses of obstetric and midwifery clinicians, obstetricians and GP obstetricians were combined to form the group ‘doctors’.

Responses for midwives and doctors were compared using Student’s *t*-test. The data was found to violate a number of assumptions of the *t*-test presumably due to the large differences in sample sizes between groups (mean difference = 225 participants). As such, random sub-samples of the midwifery group were generated and analysed. The results did not differ from those with the full sample of midwife participants and as such, the original sample is reported here. A Bonferroni adjustment of .008 was used to control for the family wise error rate associated with the use of multiple comparisons [14].

## Results

### Participants

Participants consisted of maternity care staff employed in both the public and private sectors. The sample consisted of 302 females and 34 males (N = 336). This included 281 (84%) midwives, 35 (10%) obstetricians and 21 (6%) GP obstetricians.

Comparisons were made with the Australian Health Practitioner Regulation Agency’s 2010–11 annual report [15] and the public address lists of obstetricians [16,17] to determine how representative the current study’s sample was in terms of practicing clinicians (i.e., midwives, obstetricians, GP obstetricians) in Queensland. Despite the higher numbers of midwives overall, obstetricians (18%) and GP obstetricians (10%) were better represented than midwives (4%).

### Findings

#### Decision-making

Maternity care providers were asked to rate their agreement with, “*In collaborative practice, working with primary carers, the final decision should always rest with the woman*” and “*Collaboration involves midwives and doctors working together but the doctor is the most competent in making the final decision*”. Overall, both midwives (*M* = 5.72, *SD* = 1.19) and doctors (*M* = 4.82, *SD* = 1.65) agreed that the final decision should always rest with the woman, however, midwives agreed significantly more, *t*(334) = −3.87, *p* < .001.

The two professional groups significantly differed in their perception of the doctor being the most competent with regards to decision making, with doctors agreeing that they were the most competent (*M* = 4.95, *SD* = 1.42) while midwives disagreed (*M* = 2.54, *SD* = 1.55), *t*(334) = 11.29, *p* < .001.

#### Women’s autonomy

Care providers were asked to rate their agreement with, “*For the safety of the baby, the maternity care team sometimes need to override the needs of the woman*” and “*Encouraging women to have more control over their childbearing compromises safety*”. Doctors (*M* = 4.89, *SD* = 1.48) agreed that the needs of the woman sometimes have to be overridden while midwives were neutral (*M* = 4.27, *SD* = 1.77), however, this difference was not significant, *t*(332) = 2.71, *p* > .008. Both groups then disagreed that woman having control over their childbearing would compromise safety. In this item, the professional groups again differed significantly in their disagreement with midwives expressing more disagreement (*M* = 2.14, *SD* = 1.30) than did doctors (*M* = 3.22, *SD* = 1.33), *t*(333) = 5.61, *p* < .001.

#### Legal accountability

Health professionals were asked to rate their agreement with, “*Legally, doctors are ultimately responsible, even in collaborative models*” and “*The current maternity care system allows all to be legally accountable for their own actions in a collaborative team*.” Midwives and doctors significantly differed in their responses to each of these items. Midwives disagreed that doctors are ultimately responsible (*M* = 2.69, *SD* = 1.57), compared to doctors who agreed (*M* = 5.75, *SD* = 1.34), *t*(334) = 15.04, *p* < .001. Midwives expressed a neutral response to the current maternity care system allowing all care providers to be legally accountable for their own actions (*M* = 4.21, *SD* = 1.61), while doctors disagreed that the maternity care system currently allows for this (*M* = 3.13, *SD* = 1.40), *t*(331) = −4.61, *p* < .001.

## Discussion

The current paper examined maternity care providers’ attitudes and beliefs towards women’s right to autonomous decision making during pregnancy and birth, and the legal responsibility of care providers for outcomes experienced by the mother and fetus. Our findings indicated that maternity care providers have a poor understanding of their own legal accountability, and the rights of the woman and her fetus. Also of note, five of the six survey items yielded significant differences in responses between midwives and doctors, indicating that attitudes and beliefs towards the legal rights of the woman, fetus, and health care professionals differ across maternity care professional groups.

### Decision making

Both professional groups indicated that they supported women’s right to autonomous decision making during pregnancy through their response to the item that the final decision should always rest with the woman. This is consistent with national Australian health care standards that state all health care consumers have the right to make decisions about the care they receive [1]. This belief however was not sustained or supported through another item where care providers either agreed or were neutral to the needs of the women being overridden for the safety of the baby.

Previous research suggests both midwives and obstetricians only support women to make the final decision about an aspect of their care when this decision is what the care provider prefers [7,8]. This research is supported by our findings that suggest maternity care providers’ conscious belief in women’s autonomy may not translate to actual practice. As such, there is a need to better understand care providers’ perceptions of women’s right to autonomous decision making under a variety of conditions (e.g., when the woman makes a choice against a care provider’s preference).

Also of note, when compared to midwives, doctors believed themselves to be the most competent at making final decisions. Such a belief may be associated with doctors believing that they are also more legally accountable (as discussed below). Although this finding relates to decision making amongst care providers, it has significant repercussions for the type of care women receive and the amount of support they are given to enable autonomous decision making. For example, this perception of power imbalance may inhibit midwives and doctors’ ability to work in collaboration with one another [18] and to thus focus on the needs of the women in order to provide woman-centred care [19]; a model of care which fosters women’s autonomous decision making [20]. Therefore, in order to facilitate women’s autonomy, it is essential that no one member of the collaborative team believes themselves to be more competent in decision making than other members.

### Women’s autonomy

Interestingly, the only item that care providers did not significantly differ on was ‘*For the safety of the baby, the maternity care team sometimes need to override the needs of the woman*.’ In most Western countries, including Australia, the law does not recognise the fetus until it begins extra-uterine life, and as such pregnant women have the right to refuse treatment even if this choice could cause the fetus harm or death (Cuttini et al., 2006; [21-23]). However, some lawmakers believe that no right is absolute and that a person’s autonomy is no exception to this [24]. To date there are no Australian cases that have tested this concept; however, there have been a number of court cases in other Western countries where a woman has been ordered to undergo an intervention (usually a caesarean) against her will for the sake of the fetus [2,24]. The English courts have accepted that a competent pregnant woman can refuse treatment even if that refusal may lead to increased harm to her or her fetus. In the case of *St George’s Healthcare NHS Trust v S; R v Collins, ex parte S [1998] 3 All ER 673* a lower court had ordered a woman to comply with her doctor’s recommendations to have a caesarean section due to preeclampsia, despite her wanting to birth naturally. The woman was then forced to undergo the court ordered caesarean section. The appeal court made it clear that the woman’s autonomy had been violated and that she had the right to refuse treatment.

Some care providers may feel that they are legally (or morally) responsible for the fetus and as such may override the needs of the women in order to assist the fetus [2]. This was supported by the current study’s finding where care providers were either unsure (midwives) or agreed (doctors) that the needs of the women should be overridden. Interestingly, care providers agreed that encouraging women to have control over their childbearing did not compromise safety. Given the previous finding demonstrated they believe they may have to override the wishes of the mother, this belief may not be upheld in circumstances where the woman’s control over her childbearing affects the fetus in a manner that care providers are not comfortable with.

### Legal accountability

Respecting women’s right to refuse medical treatment may be especially difficult for care providers if they feel they are legally responsible for the outcomes of the woman’s decision. This may be particularly the case for doctors, with the current study finding that doctors believed themselves to be ultimately responsible legally. This is despite the legal position that all health practitioners working in a team are legally accountable for their own negligent acts or omissions (*Elliott v Bickerstaff* [1999] NSWCA 453). Furthermore, if a woman has been given all of the information necessary to give informed consent and there is an adverse outcome that is not caused by practitioner negligence, the practitioner cannot be held liable. Thus, all health care professionals within a collaborative maternity care team are each responsible only for the care they provide―not the care provided by others. Our findings further demonstrate that care providers are poorly informed about this subject with midwives unsure about whether the current maternity care system allows for all to be held accountable for their own actions and doctors believing that it does not.

### Strengths and limitations

This study was limited in that survey responses may have been subject to response bias if care providers answered questions in a way that they felt was most politically or socially acceptable rather than what they actually believed. Furthermore, as this survey was voluntary, care providers’ responses may not be representative of all Australian maternity care providers.

#### Implications for practice and future research

Our findings have significant implications for practice and future research. There is clear ambiguity around clinicians’ understanding and beliefs of women’s autonomy and the rights of the fetus. It is essential that differences in beliefs around women’s autonomy and legal accountability between midwives and doctors are recognised in order for these health professionals to work effectively in a collaborative team [18]. Furthermore, some care providers may need to be supported to reflect on how aspects of woman-centred care may conflict with their broader values and beliefs on the rights of the fetus, and the legal and regulatory responsibilities of health professionals. Finally, there is a clear need to develop guidelines that provide information to care providers around rights to request and refuse medical treatment (particularly when women choose care outside of professional advice), and to provide policy direction on how these concepts can be applied in evidence-based, woman-centred care.

## Conclusion

Women’s right to make autonomous decisions about their care during pregnancy and birth are inconsistently supported by maternity care stakeholders. This is further complicated by the poorly informed beliefs regarding legal accountability of care providers for outcomes experienced in pregnancy and birth. This study found that both midwives and doctors were inconsistent in their responses to women being the final decision-maker on the care they receive. Furthermore, midwives and doctors differed in their attitudes and beliefs around women’s rights, and their legal responsibilities to the mother and the fetus.

## Competing interests

The authors declare that they have no competing interests.

## Authors' contributions

SK created the survey instrument in collaboration with another researcher, contributed to the conception and design of the paper, and was involved in drafting and revising the manuscript. KY contributed to the design of the paper, conducted the data analyses, and drafted the manuscript. BJ and AC contributed to the conception, development and revision of the paper. All authors read and approved the final manuscript.

## Pre-publication history

The pre-publication history for this paper can be accessed here:

http://www.biomedcentral.com/1471-2393/13/84/prepub

## Supplementary Material

Additional file 1 T-test results comparing midwives and doctors’ responses for each survey item. Click here for file
