# Supplementary material for: Maternity care providers’ perceptions of women’s autonomy and the law
Source: BMC Pregnancy Childbirth. 2013 Apr 4;13:84. doi: 10.1186/1471-2393-13-84 (PMC3668159; doi:10.1186/1471-2393-13-84)
Supplement: Additional file 1 — T-test results comparing midwives and doctors’ responses for each survey item. [file 1471-2393-13-84-S1.doc]

Collaboration in Queensland Maternity Care

Your Thoughts

Particular statements in this survey have been obtained from a pilot survey conducted by the Queensland Centre for Mothers and Babies. Some of these comments may seem extreme, and there are likely to be some you disagree with, but they do represent the opinions of some participants in the pilot study. It is important to validate the opinions we received in the pilot study, as well as conclusions drawn from the research and policy literature, on a large and representative sample of maternity care professionals in Queensland. Thus, we appreciate your participation in this study. We would like you to give us your opinion about each of these statements. Please rate the following statements by marking the response that most closely resembles your opinion, where 1 = Strongly Disagree, 4 = Neither Disagree or Agree, and 7 = Strongly Agree.

Thank you very much for your help.

Defining Collaborative Practice

A01 Please read the following statement

The National Health and Medical Research Council (NHMRC) has created a working definition of collaboration*:

*Collaborative maternity care involves collaboration* ***with*** *the woman (respecting her physical, emotional, social and cultural needs) and* ***for*** *the woman (creating a safe, high-quality, evidence-based and woman-centred environment). Collaborating clinicians are aware of, appreciate and respect each other’s skills, experience, roles and scope of practice — both as individuals and as members of professional disciplines. Collaborating clinicians communicate and cooperate, valuing opportunities to continuously improve services through education, training, review and audit. Collaborative maternity care is grounded in continuity of care and is adaptable to the local context.*

|  | | **Strongly Disagree** | **Disagree** | **Slightly Disagree** | **Neither Disagree or Agree** | **Slightly Agree** | **Agree** | **Strongly Agree** |
| --- | --- | --- | --- | --- | --- | --- | --- | --- |
| A01 | Do you agree with the NHMRC definition for use in defining collaborative maternity care in Queensland? | 1 | 2 | 3 | 4 | 5 | 6 | 7 |

A02 Please comment on what you find particularly useful about this definition (if anything), and how you would change it (if you would).

____________________________________________________________________________________________________________________________________________________________________________________________________________________________________________________________________________

____________________________________________________________________________________________________________________________________________________________________________________________________________________________________________________________________________

*NHMRC (National Health and Medical Research Council) (2009). National Guidance on Collaborative Maternity Care: Report on the Forum. NHMRC, Canberra.

| *The published literature has suggested many benefits to collaborative health care. Do you believe the following benefits result from high-quality collaboration in maternity care?* | | | | | | | | |
| --- | --- | --- | --- | --- | --- | --- | --- | --- |
|  | | **Strongly Disagree** | **Disagree** | **Slightly Disagree** | **Neither Disagree or Agree** | **Slightly Agree** | **Agree** | **Strongly Agree** |
| A03 | Improved maternal and neonatal outcomes | 1 | 2 | 3 | 4 | 5 | 6 | 7 |
| A04 | Increased efficiency of maternity care | 1 | 2 | 3 | 4 | 5 | 6 | 7 |
| A05 | Benefits for maternity care professionals on a *professional* level | 1 | 2 | 3 | 4 | 5 | 6 | 7 |
| A06 | Benefits for maternity care professionals on a *personal* level | 1 | 2 | 3 | 4 | 5 | 6 | 7 |

Current Workplace Practice

*One aim of this survey* is to explore actual current workplace practice and whether behaviour by members in your workplace is collaborative. Please rate your agreement (or disagreement) with the following statements.

|  | | **Strongly Disagree** | **Disagree** | **Slightly Disagree** | **Neither Disagree or Agree** | **Slightly Agree** | **Agree** | **Strongly Agree** |
| --- | --- | --- | --- | --- | --- | --- | --- | --- |
| A07 | The woman is an equal contributor to the collaborative team | 1 | 2 | 3 | 4 | 5 | 6 | 7 |
| A08 | There is respect in the capabilities of both medical and midwifery professions | 1 | 2 | 3 | 4 | 5 | 6 | 7 |
| A09 | There is trust in the capabilities of both medical and midwifery professions | 1 | 2 | 3 | 4 | 5 | 6 | 7 |
| A10 | Midwives and doctors work together to achieve the best possible outcomes for childbearing women | 1 | 2 | 3 | 4 | 5 | 6 | 7 |
| A11 | Maternity care professionals do not always communicate openly with each other | 1 | 2 | 3 | 4 | 5 | 6 | 7 |
| A12 | I am a valued member of the team | 1 | 2 | 3 | 4 | 5 | 6 | 7 |
| A13 | Generally, I find it difficult to exchange ideas easily with midwifery staff | 1 | 2 | 3 | 4 | 5 | 6 | 7 |
| A14 | Generally, I find it difficult to exchange ideas easily with medical staff | 1 | 2 | 3 | 4 | 5 | 6 | 7 |
| A15 | I share decision-making with other members of the maternity care team | 1 | 2 | 3 | 4 | 5 | 6 | 7 |
| A16 | Interprofessional social relationships outside of work are important | 1 | 2 | 3 | 4 | 5 | 6 | 7 |
| A17 | I respect the professional decision-making and skills of midwives I work with | 1 | 2 | 3 | 4 | 5 | 6 | 7 |
| A18 | I respect the professional decision-making and skills of doctors I work with | 1 | 2 | 3 | 4 | 5 | 6 | 7 |
| A19 | Midwives are routinely involved in formal interprofessional review of adverse events | 1 | 2 | 3 | 4 | 5 | 6 | 7 |

How Does Collaboration Work For You?

*Please remember that certain statements below represent views obtained from pilot participants. It is likely that you will agree with some but disagree with other statements. To allow us to determine the prevalence of these comments in a broad population of maternity care professionals, please state your level of agreement with each statement. This section relates specifically to research that suggests professional groups in maternity care often have a different understanding of ‘collaboration’.*

| ***Medical models of care*** | | | | | | | | | | | | |  |
| --- | --- | --- | --- | --- | --- | --- | --- | --- | --- | --- | --- | --- | --- |
|  | | | | **Strongly Disagree** | | **Disagree** | | **Slightly Disagree** | **Neither Disagree or Agree** | **Slightly Agree** | **Agree** | **Strongly Agree** |  |
| A20 | Collaboration involves midwives and doctors working together but the doctor is most competent in making the final decision | | | 1 | | 2 | | 3 | 4 | 5 | 6 | 7 |  |
| A21 | Low-risk women should see a doctor at least once in their pregnancy | | | 1 | | 2 | | 3 | 4 | 5 | 6 | 7 |  |
| A22 | Doctors should review all women in labour | | | 1 | | 2 | | 3 | 4 | 5 | 6 | 7 |  |
| A23 | Most women believe doctors are ultimately responsible, even in collaborative models | | | 1 | | 2 | | 3 | 4 | 5 | 6 | 7 |  |
| A24 | Legally, doctors are ultimately responsible, even in collaborative models | | | 1 | | 2 | | 3 | 4 | 5 | 6 | 7 |  |
| A25 | Women should only experience labour and birth in a place where anaesthetic and surgical facilities are available on site | 1 | | | 2 | | 3 | | 4 | 5 | 6 | 7 |  |
| A26 | Generally speaking, doctors provide women-centred care | 1 | | | 2 | | 3 | | 4 | 5 | 6 | 7 |  |
|  | | | | | | | | | | | | | |
| ***Midwifery models of care*** | | | | | | | | | | | | | |
|  | | | | **Strongly Disagree** | | **Disagree** | | **Slightly Disagree** | **Neither Disagree or Agree** | **Slightly Agree** | **Agree** | **Strongly Agree** |  |
| A27 | Women in all risk categories should be able to receive continuous care from a known midwife | | | 1 | | 2 | | 3 | 4 | 5 | 6 | 7 |  |
| A28 | A doctor does not need to be involved in a birth which is progressing normally | | | 1 | | 2 | | 3 | 4 | 5 | 6 | 7 |  |
| A29 | Obstetricians should care for high-risk or complicated pregnancies only | | | 1 | | 2 | | 3 | 4 | 5 | 6 | 7 |  |
| A30 | Midwives have the skills to provide safe care as the primary carer for women identified with no risk factors | | | 1 | | 2 | | 3 | 4 | 5 | 6 | 7 |  |
| A31 | Reducing Australian rates of interventions will improve maternal outcomes | | 1 | | | 2 | | 3 | 4 | 5 | 6 | 7 |  |
| A32 | Reducing Australian rates of interventions will improve infant outcomes | | 1 | | | 2 | | 3 | 4 | 5 | 6 | 7 |  |
|  | | | | | | | | | | | | | |
| ***Delivering woman-centred care*** | | | | | | | | | | | | | |
|  |  | | | **Strongly Disagree** | | **Disagree** | | **Slightly Disagree** | **Neither**  **Disagree or**  **Agree** | **Slightly**  **Agree** | **Agree** | **Strongly**  **Agree** |  |
| A33 | In collaborative practice, working with primary carers, the final decision should always rest with the woman | | | 1 | | 2 | | 3 | 4 | 5 | 6 | 7 |  |
| A34 | Traditional models of care and hospital policies result in the woman often not the focus of care | | | 1 | | 2 | | 3 | 4 | 5 | 6 | 7 |  |
| A35 | For the safety of the baby, the maternity care team sometimes need to override the needs of the woman | | | 1 | | 2 | | 3 | 4 | 5 | 6 | 7 |  |
| A36 | Encouraging women to have more control over their childbearing compromises safety | | | 1 | | 2 | | 3 | 4 | 5 | 6 | 7 |  |
| A37 | Maternity care professionals require guidelines for women who choose birthing options that are not appropriate to level of risk | | | 1 | | 2 | | 3 | 4 | 5 | 6 | 7 |  |

Factors Affecting Collaborative Practice

*Please remember that certain statements below represent views obtained from pilot participants. It is likely that you will agree with some but disagree with other statements. To allow us to determine the prevalence of these comments in a broad population of maternity care professionals, please state your level of agreement with each statement. This section relates specifically to elements that have been identified by maternity care professionals and researchers to affect the collaboration process.*

| ***The current maternity care system:*** | | | | | | | | |  |
| --- | --- | --- | --- | --- | --- | --- | --- | --- | --- |
|  | | **Strongly Disagree** | **Disagree** | **Slightly Disagree** | **Neither Disagree or Agree** | **Slightly Agree** | **Agree** | **Strongly Agree** |  |
| A38 | Encourages maternity care professionals to work collaboratively | 1 | 2 | 3 | 4 | 5 | 6 | 7 |  |
| A39 | Has inconsistent policies, procedures, and guidelines regarding collaboration | 1 | 2 | 3 | 4 | 5 | 6 | 7 |  |
| A40 | Fosters managerial support for collaborative practice | 1 | 2 | 3 | 4 | 5 | 6 | 7 |  |
| A41 | Provides adequate support to allow equal and appropriate contribution to collaborative practice | 1 | 2 | 3 | 4 | 5 | 6 | 7 |  |
| A42 | Does not provide payment schedules to maternity care professionals that cultivate appropriate contribution to collaborative practice | 1 | 2 | 3 | 4 | 5 | 6 | 7 |  |
| A43 | Has time structures in place to allow collaboration between maternity care professionals to occur | 1 | 2 | 3 | 4 | 5 | 6 | 7 |  |
| A44 | Provides adequate funding to support collaboration in my workplace | 1 | 2 | 3 | 4 | 5 | 6 | 7 |  |
| A45 | Cultivates a culture non-conducive to collaborative practice | 1 | 2 | 3 | 4 | 5 | 6 | 7 |  |
| A46 | Allows all to be legally accountable for their own actions in a collaborative team | 1 | 2 | 3 | 4 | 5 | 6 | 7 |  |

| ***Referral between professionals and guidelines*** | | | | | | | | |  |
| --- | --- | --- | --- | --- | --- | --- | --- | --- | --- |
|  | *Are you familiar with the following guidelines:* |  |  |  |  |  | **Yes** | **No** |  |
| A47a | Royal Australian and New Zealand College of Obstetricians and Gynaecologists (RANZCOG) Guideline: Suitability Criteria for Models of Care and Indications for Referral within & between Models of Care | | | | |  | 1 | 2 |  |
| A47b | Australian College of Midwives (ACM) National Midwifery Guidelines for Consultation and Referral | | | | |  | 1 | 2 |  |
| Where you are familiar with the set of guidelines, please indicate your agreement (or disagreement) with each statement below (please mark “not applicable” if you are not familiar with a set of guidelines). | | | | | | | | | |
|  | | **Strongly Disagree** | **Disagree** | **Slightly Disagree** | **Neither Disagree or Agree** | **Slightly Agree** | **Agree** | **Strongly Agree** | **Not Applicable** |
| A48 | The RANZCOG guidelines are appropriate for use in Australia | 1 | 2 | 3 | 4 | 5 | 6 | 7 | 8 |
| A49 | The ACM guidelines are appropriate for use in Australia | 1 | 2 | 3 | 4 | 5 | 6 | 7 | 8 |
| A50 | New guidelines would be more appropriate for referral of women in Australia | 1 | 2 | 3 | 4 | 5 | 6 | 7 | 8 |

A50a If you indicated that new guidelines would be more appropriate, what you find particularly useful in new guidelines, or what would you change about old guidelines.

____________________________________________________________________________________________________________________________________________________________________________________________________________________________________________________________________________

___________________________________________________________________

| ***Barriers to collaboration*** | | | | | | | | |  |
| --- | --- | --- | --- | --- | --- | --- | --- | --- | --- |
| *Please note:* *Participants in the pilot study indicated a number of barriers to high-quality collaboration, including contested areas (“turf wars”) between midwives and doctors. Please rate your agreement with the following statements taken from the pilot feedback and other published literature. You may find some of these statements extreme, so we appreciate you giving us your views.* | | | | | | | | |  |
|  | | **Strongly Disagree** | **Disagree** | **Slightly Disagree** | **Neither Disagree or Agree** | **Slightly Agree** | **Agree** | **Strongly Agree** |  |
| A51 | “Collaboration fails due to ‘turf wars’” | 1 | 2 | 3 | 4 | 5 | 6 | 7 |  |
| A52 | “Doctors are being called late, barred from rooms and left to deal with the ‘train wrecks’” | 1 | 2 | 3 | 4 | 5 | 6 | 7 |  |
| A53 | “There has been an isolation of medical staff due to mistrust” | 1 | 2 | 3 | 4 | 5 | 6 | 7 |  |
| A54 | “There is a culture of bullying, disrespect, and resentment between obstetricians and midwives” | 1 | 2 | 3 | 4 | 5 | 6 | 7 |  |
| A55 | “There is historical animosity between doctors and midwives” | 1 | 2 | 3 | 4 | 5 | 6 | 7 |  |
| A56 | “Midwives need to stop being so precious about seeing themselves as the only people capable of providing woman centred care” | 1 | 2 | 3 | 4 | 5 | 6 | 7 |  |
| A57 | “Respectful relationships between maternity care professionals are difficult to develop” | 1 | 2 | 3 | 4 | 5 | 6 | 7 |  |
| A58 | “Interprofessional groups do not work because one profession usually dominates the proceedings” | 1 | 2 | 3 | 4 | 5 | 6 | 7 |  |
| A59 | “Collaboration does not work because doctors dominate decision-making” | 1 | 2 | 3 | 4 | 5 | 6 | 7 |  |
| A60 | “Changes towards more collaboration in maternity services will undermine the excellent obstetric safety record in Australia” | 1 | 2 | 3 | 4 | 5 | 6 | 7 |  |
| A61 | “More collaborative care would exclude junior doctors from attending enough normal births” | 1 | 2 | 3 | 4 | 5 | 6 | 7 |  |
| A62 | “General practitioners should advise women of all options for their maternity care, including private practice midwives and midwifery models of care” | 1 | 2 | 3 | 4 | 5 | 6 | 7 |  |
| A63 | “Private practice midwives should provide opportunities for woman to meet members of the team in the antenatal period; the people who are responsible for care in the event of unexpected complications” | 1 | 2 | 3 | 4 | 5 | 6 | 7 |  |

Professional Values and Beliefs

*Please note:* These questions relate specifically to maternity care professionals’ attitudes towards birth and the perceived world views of other maternity care professionals. There is research evidence suggesting that differences in the world views and attitudes of maternity care professionals can impede the process of collaboration. Please help us assess whether these findings apply in Queensland by indicating your agreement (or disagreement) with each statement.

| ***Successful collaboration requires:*** | | | | | | | | | |
| --- | --- | --- | --- | --- | --- | --- | --- | --- | --- |
|  | | **Strongly Disagree** | **Disagree** | **Slightly Disagree** | **Neither Disagree or Agree** | **Slightly Agree** | **Agree** | **Strongly Agree** |  |
| A64 | There is no role for private midwives in Australia | 1 | 2 | 3 | 4 | 5 | 6 | 7 |  |
| A65 | There is no role for private obstetricians in Australia | 1 | 2 | 3 | 4 | 5 | 6 | 7 |  |
| A66 | Generally, midwives tend to understate the risks involved in pregnancy and birth | 1 | 2 | 3 | 4 | 5 | 6 | 7 |  |
| A67 | Generally, doctors tend to overstate the risks involved in pregnancy and birth | 1 | 2 | 3 | 4 | 5 | 6 | 7 |  |
| A68 | Doctors and midwives share the same values and beliefs around maternity care | 1 | 2 | 3 | 4 | 5 | 6 | 7 |  |
| A69 | Doctors and midwives generally agree on the best way to manage the care of women with *uncomplicated* pregnancies | 1 | 2 | 3 | 4 | 5 | 6 | 7 |  |
| A70 | Midwives and doctors generally agree on the best way to manage the care of women with *complicated* pregnancies | 1 | 2 | 3 | 4 | 5 | 6 | 7 | **Not Applicable** |
| A71 | As a midwife, I am interested in accessing the MBS and PBS when available | 1 | 2 | 3 | 4 | 5 | 6 | 7 | 8 |
| A72 | As a doctor, I am interested in working with midwives who access the MBS and PBS when available | 1 | 2 | 3 | 4 | 5 | 6 | 7 | 8 |

Collaborative Practice In Queensland

Shared definitions of “collaboration”, “woman-centred care”, along with open communication, respect and trust between professionals have been identified as important in enhancing collaborative practice. The literature suggests that other variables are also important. Please rate your agreement (or disagreement) about the importance of the variables in this section.

|  | | **Strongly Disagree** | **Disagree** | **Slightly Disagree** | **Neither Disagree or Agree** | **Slightly Agree** | **Agree** | **Strongly Agree** |
| --- | --- | --- | --- | --- | --- | --- | --- | --- |
| A73 | Individual staff members who are confident and self aware | 1 | 2 | 3 | 4 | 5 | 6 | 7 |
| A74 | Willingness to collaborate | 1 | 2 | 3 | 4 | 5 | 6 | 7 |
| A75 | Ongoing commitment to collaboration | 1 | 2 | 3 | 4 | 5 | 6 | 7 |
| A76 | Structured information sharing (i.e. case review with all staff) | 1 | 2 | 3 | 4 | 5 | 6 | 7 |
| A77 | Unstructured information sharing (i.e. morning teas, informal telephone calls) | 1 | 2 | 3 | 4 | 5 | 6 | 7 |
| A78 | Social activities with all staff | 1 | 2 | 3 | 4 | 5 | 6 | 7 |
| A79 | A clear process for resolving disagreements and conflicts | 1 | 2 | 3 | 4 | 5 | 6 | 7 |
| A80 | Working together to provide optimal care whilst taking individual responsibility for own actions | 1 | 2 | 3 | 4 | 5 | 6 | 7 |
| A81 | Each member of the team being accountable for their own actions | 1 | 2 | 3 | 4 | 5 | 6 | 7 |
| A82 | Increased joint education between doctors and midwives | 1 | 2 | 3 | 4 | 5 | 6 | 7 |

A83 What other conditions would enhance your collaboration with other maternity care professionals (if any)?

____________________________________________________________________________________________________________________________________________________________________________________________________________________________________________________________________________

______________________________________________________________________________________________________________________________________

____________________________________________________________________________________________________________________________________________________________________________________________________________________________________________________________________________

______________________________________________________________________________________________________________________________________

Demographic Information

**D01 Sex:** □ Female □ Male

**D02 Age Group:** □ 24 and under □ 25-34 □ 35-49 □ 50-65 □ 66 and over

**D03 What is your highest level of education?**

□ Certificate

□ Diploma

□ Bachelor degree

□ Masters degree

□ PhD or Professional Doctorate

□ Other (please specify) ______________________

**D04 What is your current qualification?**

□ Medical Student

□ Midwifery Student

□ Qualifications in Midwifery

□ Qualifications in Midwifery and Nursing

□ Obstetric Registrar

□ Obstetrician

□ General Practitioner (GP)

□ GP Obstetrician (DRANZCOG)

□ GP Advanced Obstetrician (DRANZCOG Advanced)

□ Other (please specify) ______________________

**D05 What is your current role?**

□ Clinical GP

□ Clinical Midwife

□ Clinical Obstetrician

□ Educator *(please answer D05a)*

□ Researcher *(please answer D05a)*

□ Maternity Services Manager *(please answer D05a)*

□ Policy Officer *(please answer D05a)*

□ Project Officer *(please answer D05a)*

□ Other (please specify) ______________________

**D05a Do you currently practice clinically?** □ Yes □ No

**D06 How many years/months have you been working in your current role(s)?**

_________ years ________ months

*Other roles:*

______________ _________ years ________ months

______________ _________ years ________ months

**D07 Which of the following maternity services do you provide?**

□ Antenatal

□ Community-based

□ Hospital-based

□ Intrapartum

□ Postnatal

□ Community-based

□ Hospital-based

□ I do not currently provide maternity care

**D08 Which of the following best describes your current work situation?**

(*Please tick all that apply)*

□ Full-time public hospital employee (*Description of levels next page)*:

- Level 1

□ Level 2

- Level 3
- Level 4
- Level 5
- Level 6

□ Part-time public hospital employee (*Description of levels next page)*:

- Level 1

□ Level 2

- Level 3
- Level 4
- Level 5
- Level 6

□ Full-time private hospital employee

□ Part-time private hospital employee

□ Private practice with visiting privileges to a hospital

□ Private practice without visiting privileges to a hospital

□ Team midwifery program

□ Case load or group practice midwifery program

□ Birth centre

□ Shared care model (GP and midwife)

□ Tertiary education sector

□ Other (please specify) ______________________________

**D09 What best describes your location?**

□ Metropolitan

□ Regional

□ Rural

□ Remote

**D10 Which Health Service District do you currently work in?**

□ Cairns and Hinterland □ Gold Coast □ South West

□ Cape York □ Mackay □ Sunshine Coast-Wide Bay

□ Central Queensland □ Metro North □ Torres Strait-Northern Peninsula

□ Central West □ Metro South □ Townsville

□ Darling Downs-West Moreton □ Mt Isa

□ Other (please specify) ________________________________

Thank you for your time and effort in completing the survey. Please use the reply paid envelope provided to return the survey. If you have mislaid the envelope, please return this survey in a normal envelope (you do not need a stamp) to:

Queensland Centre for Mothers and Babies (692)

The University of Queensland

Reply Paid 6469

St Lucia QLD 4067

Levels of Care

Clinical Services Capability Framework (CSCF) Description

**Level 1** - no planned births or maternity inpatient services, and provides community antenatal and/or postnatal care for women and infants. Care is provided in partnership with higher level services.

**Level 2** - primarily delivered by midwives and medical practitioners. An obstetrician may provide consults for the health service. Essentially this service provides antenatal and postnatal care for women and infants without identified risk factors.

**Level 3** - provides community and inpatient care for antenatal and postnatal women and neonates without identified risk factors. This level service will have planned birth care for healthy women with a pregnancy of 37 weeks gestation or more who are not expected to have labour or birth complications. Elective and emergency caesarean birth can be performed on-site within the service capability.

**Level 4** - capable of providing maternity care for low and moderate risk women, but does not have the level of service to care for women with complex high risk conditions. A Level 4 service will have a multidisciplinary maternity staff, and offer a number of maternity models of care including provision of or referral to midwifery community care.

**Level 5** - provides multidisciplinary care for low to high risk pregnancies and can undertake invasive antenatal diagnostic procedures. Core service provision includes close monitoring and early intervention by trained obstetricians and midwives, neonatologists or paediatricians and neonatal nurses and obstetric physicians.

**Level 6** - provides all levels of care including the highest level of complex care for women with serious obstetric and fetal conditions requiring high level multidisciplinary care.
